# GB virus-C – a virus without a disease: We cannot give it chronic fatigue syndrome

**DOI:** 10.1186/1471-2334-5-78

**Published:** 2005-09-28

**Authors:** James F Jones, Prasad S Kulkarni, Salvatore T Butera, William C Reeves

**Affiliations:** 1 Viral Exanthems and Herpesvirus Branch, Division of Viral and Rickettsial Diseases, Centers for Disease Control and Prevention, 1600 Clifton Rd, mailstop A-15, Atlanta, Georgia, 30333, USA; 2 Laboratory Branch, Division of HIV/AIDS Prevention, Centers for Disease Control and Prevention, 1600 Clifton Rd, mailstop G-19, Atlanta, Georgia, 30333, USA

## Abstract

**Background:**

Chronic fatigue syndrome (CFS) is an illness in search of an infectious etiology. GB virus-C (GBV-C) virus is a flavivirus with cell tropism and host defense induction qualities compatible with a role in producing the syndrome. The GBV-C genome is detectable in 4% of the population and 12% of the population is seropositive. The present study evaluated the association between infection with GBV and CFS.

**Methods:**

We used a commercial EIA to detect antibodies against the GBV-C E2 protein and a quantitative real-time RT-PCR assay to detect active GBV-C infection. Sera were from a case control study of CFS in Atlanta, Georgia. The Fisher's exact two-tailed test was used for statistical analysis.

**Results:**

Two of 12 CFS patients and one of 21 controls were seropositive for prior GBV-C infection and one control had viral RNA detected, indicating active infection. The results are not statistically different.

**Conclusion:**

We found no evidence that active or past infection with GBV is associated with CFS.

## Background

Chronic fatigue syndrome (CFS) affects 400,000 to 900,000 adults in the United States [1,2]. At least a quarter of those suffering from CFS are unemployed or receiving disability because of the illness; the average affected family forgoes $20,000 annually in lost earnings and wages (approximately half of the average United States household income); and, the annual value of lost productivity in the United States is approximately $9 billion [2-4]. Despite the public health burden imposed by CFS, diagnostic, treatment and prevention strategies have proven difficult to devise because the etiology, pathophysiology and risk factors of CFS remain unknown [reviewed in [5]].

Because the symptoms resemble those of infectious diseases and because CFS may follow an acute infectious disease, a considerable body of work has attempted to identify persistent or reactivated infection in patients with this illness [reviewed in [5]]. These efforts have included analysis of seroprevalence, evaluation of viral RNA and DNA (i.e., latent/active infection), and most recently, molecular identification procedures for unique, previously uncharacterized pathogens [6-12]. None of these reports have developed convincing evidence for a significant association between any infectious agent and CFS.

However, negative, inconclusive, and conflicting case control studies do not mean that infectious etiologies for CFS should be dismissed. A recently discovered flavivirus, GB virus-C/HGV (GBV-C) [13-17], has many properties that demanded a study assessing its possible association with CFS. GBV-C preferentially replicates in peripheral blood mononuclear cells (PBMC), primarily B and T lymphocytes, and in bone marrow *in vivo *[18,19]. Of note, some flaviviruses activate the classic complement pathway [20] and we have found a high prevalence of split products (C3a, C4a, and C5a) in patients with CFS compared to controls [21]. We have also examined mononuclear cell populations for gene expression patterns and have found differences between patients and control subjects [22,23]. GBV-C viremia may persist for several years following primary infection and serologically antibodies are generated against the envelope protein, E2 [reviewed in [15,18]]. The antibodies are also long-lived and may protect against re-infection [24]. GBV-C viremia and presence of antibodies are usually mutually exclusive, and only a small percentage of exposed individuals exhibit both viremia and anti-E2 antibody [25]. As yet, no illness has been associated with GBV infection.

Therefore, thinking that complement activation and altered mononuclear cell gene expression might reflect continued infection with this virus, we chose to evaluate banked serum specimens from subjects enrolled in a case control study for GBV-C RNA, an indication of persistent active infection that might be associated with CFS and for specific antibodies associated with clearance of a GBV-C infection (anti-E2) as an indicator of past infection with this virus.

## Methods

This study adhered to human experimentation guidelines of the U.S. Department of Health and Human Services and the Helsinki Declaration. The CDC Institutional Review Board approved study protocols. All participants were volunteers who gave informed consent.

### Study subjects

Subjects and study design have been presented in detail elsewhere [25,26]. In brief, 26 patients with CFS (23 women and 3 men) were recruited from a physician surveillance study in Atlanta [26]. Patients met the 1988 CFS research case definition [27] and had been ill for no more than 10 years. Two age-(± 5 years), race-, and sex-matched nonfatigued control subjects were contacted by means of random-digit dialing in the Atlanta area. Subjects' clinical, seroepidemiologic [6], and immunological characteristics have been reported [28]. For the current pilot study, testable sera were available from 12 cases and 21 controls.

### Laboratory procedures

#### Detection of GBV-C infection

Plasma samples were tested for antibodies against the GBV-C E2 envelope protein by a μPLATE Anti-HGenv microtiter assay (Roche Diagnostics Corp., Indianapolis, Ind.). Furthermore, nucleic acids were extracted from 200 μL of plasma by using the QIAamp Viral RNA Mini Kit (Qiagen Inc., Valencia, Calif.), as per the manufacturer's recommendations. A real-time quantitative reverse-transcriptase polymerase chain reaction (qRT-PCR) was developed to detect GBV-C and determine the GBV-C viral load. Five microliters of extracted nucleic acids were used in the qRT-PCR assay, performed in a final volume of 50 μL using the Quantitect Probe RT-PCR kit (Qiagen). The primers used were: GBV-C 03.1-F – 5' GCACGGTCCACAGGTGTT 3' (nucleotides 226-243 of the sequence with GenBank accession number U44402[14] and GBV-C 03.2-R – 5' GTACGTGGGCGTCGTTTG 3' (nucleotides 313–330). The probe used had the sequence 5' CCGACGTCAGGCTCGTCGTTAAAC 3' (nucleotides 268–291), and was labeled with 6-carboxy-fluorescein (FAM) on the 5' end and a dark quencher on the 3' end. This combination of primers resulted in an amplicon of 105 base pairs (bp). Serial dilutions of *in vitro *transcribed RNA generated from a linearized plasmid encoding nucleotides 136–400 of the 5' UTR of GBV-C with GenBank accession number U44402[14] was used as a quantitative standard curve.

### Statistics

Based on the estimated prevalence of GBV-C (< 4%) in healthy blood donors in the US [29], the available sample size of 12 CFS subjects and 21 controls should allow detection of 60 % positivity in viremia or positive serology at the 95% confidence level with a power of 80: with at least 50% of persons with CFS demonstrating viremia. These estimates are based on the assumption that if GBV-C infection is the primary cause CFS, the majority of CFS subjects would be positive in one of the assay systems. Results were analyzed using Fisher's exact test (2-tailed). Odds ratios were calculated from a Chi square table and confidence intervals defined using the Wald statistic.

## Results and discussion

Two cases (16.7%) and 1 control (4.8%) were seropositive (2-tailed Fisher's exact test p = .54). Only one person (a control subject) had detectable GBV-C RNA. Although the odds ratio of this difference is 5.14, the confidence interval (CI 0.32–49.59) demonstrates the difference was statistically insignificant.

This exploratory study did not identify significant differences in active or cleared GBV-C infections between individuals fulfilling a clinical definition of CFS or in control subjects. These data would not exclude GBV-C infection in small percentages (<20%) of CFS patients.

Since previous studies that used seropositivity or gene expression alone have failed to demonstrate an association between specific infections and CFS, we reasoned that a combined serological and virological approach would be more appropriate since only ongoing infection with GBV-C is associated with viremia. These results are consistent with values reported in a variety of population studies regarding the prevalence of GBV-C. Previous studies have addressed *Herpesviridae*, enteroviruses, retroviruses, hepatitis C, HTLV-II [5] and recently parvovirus [30] and Borna virus [31], but no definitive causative links have been made. Thus, another attempt to link a majority of CFS cases with a specific infectious agent did not identify a specific association.

## Conclusion

CFS remains a clinically identifiable, but daunting, medical and psychological problem.

## Competing interests

The author(s) declare that they have no competing interests

## Authors' contributions

JJ and SB conceived the study. JJ was responsible for specimen identification, evaluation of results and preparation of the manuscript. PK performed the antibody and PCR procedures and participated in the preparation of the manuscript. SB supervised the laboratory procedures and participated in the preparation of the manuscript. WR was responsible for the primary patient study and participated in the design and evaluation of the study, and participated in the preparation of the manuscript.

## Pre-publication history

The pre-publication history for this paper can be accessed here:


